# Examining the Role of Islamic Moral Gradation in Contemporary Vaccination Practices

**DOI:** 10.7759/cureus.85604

**Published:** 2025-06-09

**Authors:** Bilal Irfan, Ibrahim Omeish, Majd Abu Alnour, Ahmad M Khleif, Basel Tarab

**Affiliations:** 1 Center for Surgery and Public Health, Brigham and Women's Hospital, Boston, USA; 2 Department of Neurology, University of Michigan Medical School, Ann Arbor, USA; 3 Center for Bioethics, Harvard Medical School, Boston, USA; 4 Department of Molecular and Cellular Biology, Harvard University, Cambridge, USA; 5 Department of Global Health Research, Horizons Academy, Gaza, PSE; 6 Internal Medicine, Hebron University, Hebron, PSE

**Keywords:** fatwa, health behavior, islamic bioethics, moral gradation, vaccinations

## Abstract

This editorial explores the evolving role of Islamic jurisprudence in addressing contemporary preventive health measures, particularly vaccination. Unlike curative interventions, preventive measures such as vaccines present unique ethical and legal dilemmas for Islamic scholars. The paper examines the limitations of fatwa reviews, emphasizing methodological challenges like publication bias, decentralized rulings, and diverse linguistic contexts. It highlights the nuances of moral gradation assigned to vaccination, analyzing whether vaccines are framed as obligations (*fardh kifayah* or *fardh ‘ayn*), encouraged (*mustahab*), or discouraged (*makrūh*), and the implications for public health strategies. The concepts of public benefit (*maṣlaḥa*) and no harm (*la darar*) are central to the ongoing discourse when aiming to potentially align vaccination with the objectives of Islamic law (*maqasid al-shariah*), which is not without contestation. There is an evaluation on some of the intersections of religious and medical ethics, questioning whether assigning moral gradation to vaccination overextends the scope of Islamic jurisprudence and the role of healthcare providers in navigating this terrain.

## Editorial

In the context of the Islamic ethico-legal discourse, the question of whether Islamic legal rulings (fatāwa), which are based on an exercise of independent reasoning (ijtihād) and are nonbinding and contextual in nature, should even address issues pertaining to preventative health measures such as vaccination, is not without contention [1]. Unlike the more straightforward guidance given for treatments addressing existing conditions, advising on preventative measures enters a new dimension, one that diverges from the definitive nature of addressing illness itself. This shift from curative to preventative guidance, and potentially assigning moral gradation to the act(s) that follow, raises a unique dilemma: should Islamic scholars weigh in on issues of prevention, which may only potentially alleviate harm? Or, rather, should they restrict their counsel to the permissibility or impermissibility of receiving a vaccine based on the contents and ingredients within it? Exploring this matter opens an opportunity to evaluate the broader role of fatwa in a domain that is still being charted, and to question if this role aligns with the core tenets of Islamic jurisprudence.

Historical jurisprudence offers several instructive precedents in which jurists adopted a forward‐leaning posture on taking action to prevent harm, thereby legitimizing scholarly engagement with vaccination today. Ottoman and Egyptian scholars issued opinions, at times after consulting physicians, on quarantine rules and the need to protect communal life. Even centuries earlier, Islamic scholars issued treatises on controlling the spread of plague. These examples reveal a jurisprudential toolkit that is both preventive and inductive, indicating that contemporary scholars who weigh the collective benefits of vaccination are not departing from, but rather extending, a well-established Islamic hermeneutic of harm reduction.

The practice of reviewing Islamic legal rulings is inherently limited due to the diverse and decentralized manner in which these rulings are issued and disseminated. Islamic legal rulings can be verbal or written, and they appear across various media, including radio and television programs, websites, newspapers, book collections, policy reports, academic papers, and can be sought individually by people from qualified scholars [1]. This diversity, combined with the multitude of languages in which Islamic legal rulings are rendered, makes it nearly impossible to comprehensively gather all relevant rulings on any given issue. Vaccinations are a newer issue, and thus consulting examples or even relevant analogies prior to the 19th and 20th centuries may be all but impossible. Reviews of Islamic legal rulings, not quite unlike systematic reviews of the literature, require a clearly delineated search strategy with relevant inclusion and exclusion criteria to ensure the relevant data is captured. It is worth noting that no central database or repository such as PubMed exists for such Islamic legal rulings. An additional challenge is the issue of publication bias [1]. Islamic legal rulings issued by prominent scholars or those that provoke controversy tend to be widely circulated and more readily accessible. This visibility does not inherently confer greater legal authority or merit to these rulings but may influence their application among Muslims simply due to their prevalence. Conversely, less prominent or regionally specific Islamic legal rulings may not gain the same traction, despite their relevance to particular contexts. This imbalance underscores the potential for skewed interpretations when fatwa reviews rely heavily on the most visible rulings. The noncomprehensive nature and publication bias of reviews of Islamic legal rulings raise important methodological concerns. For example, there are key differences between Islamic legal rulings issued by individual jurists, those by juridical councils following extensive deliberations, and those originating from scholars adhering to the methodologies of different schools of Islamic jurisprudence. Drawing definitive conclusions based solely on the numerical count of Islamic legal rulings, as illustrated here, can be misleading. Such reviews of Islamic legal rulings are more akin to qualitative research, where the value lies in thematic analysis rather than in quantitative prevalence. The rigor of the arguments presented within Islamic legal rulings, as well as the precision with which biomedical contexts are articulated, is far more critical in evaluating their relevance and applicability [1]. The didactic nature of the issuance of these rulings is also important, whether the jurist(s) consulted applicable physicians, researchers, or experts in the field, as in the case of vaccinations, to fully understand the properties and mechanisms at play that can impact the verdict. Thus, it is imperative to note that finding a numerical majority of rulings permitting or prohibiting a medical practice does little to advance either position without a robust examination of the reasoning behind them.

With the context laid out above regarding the inherent limitations of simply reviewing rulings issued by religious figures and scholars, it is worth considering what has been commented about as it pertains to the assigning of a moral status to the act of receiving a vaccine, or lack thereof. The essential questions that form are what type of gradation has been assigned, if any, i.e. has it been postulated by scholars that the act of receiving a vaccine is liked or encouraged (mustahab), or on the inverse disliked or prohibitively disliked or discouraged (makrūh), or even forbidden (ḥarām) if not taken. This is a distinct subsidiary point, though related to, the general discourse of the use of vaccinations being potentially under the objectives of Islamic law (maqasid al-shari'a), which aims to protect five categories, spanning religion (al-dîn), life (al-nafs), capacity (al-‘aql), progeny (al-nasl), and property (al-mâl) [2]. The prioritization of public welfare over individual interests is often involved in matters concerning societal health and well-being. At times, the Islamic concept of public benefit (maṣlaḥa) and, in the case of vaccinations particularly, no harm (la darar) are articulated by scholars to position that the protection of life is not only an essential goal, but that in cases of conflict it should outweigh individual preferences. To postulate an opinion on a matter is to undergo the process of juridical reasoning (ijtihād), which merits 'reward' in the Islamic moral framework. Intention is also a crucial framing in receiving reward for an act, and a morally righteous deed can be classified as such based on its intention. For example, choosing to receive medication with the intention of maintaining the body's health to do other ritualistic acts such as prayer, or to work and provide for one's family as ordained to please God, or to act in Islamically-sound manners, would allow the act of receiving the medication itself be a cause of reward. Associating positive reward with the sheer act of taking medication, especially if coupled with intentions to be able to perform other acts as stated above, or to model the example of the Prophet, can be a source of moral reward. Such pronouncements lend religious legitimacy to medical interventions and may serve to encourage Muslims to pursue measures that preserve health [3]. This is distinct, though, from the debates surrounding whether scholars should delve into promoting preventive actions like vaccination as a proactive step, potentially outside their conventional role, or assigning moral status to it. 

When navigating modern health interventions, Islamic scholars often rely on the concepts of dire necessity (ḍarūra) and extreme need (ḥājah), which allow for some limited leniency in otherwise prohibited acts to prevent harm. In cases where there is a high probability (ghalabat al-ẓann) of therapeutic benefit, such as a vaccine's effectiveness, these principles may be leveraged to support recommendations for its use. This may also come up in the issue of the ingredients of a certain medication. Such frameworks create room within the confines of Islamic law to accommodate preventive measures aimed at protecting life, with the concept that what is necessary to fulfill an obligation is itself obligatory (maa laa yatimm al-wajib illa bihi fa huwa wajibun) being potentially invoked to underscore the necessity of vaccinations as a means to preserve life, a fundamental Islamic obligation. This is not without contention, and if one were to tie the reception of vaccination to being a fundamental obligation, as above, then it may incur some form of unlikability, at the minimum, to not take it [4].

In issuing rulings endorsing vaccination, jurists walk a fine line between guiding Muslims on preventive health measures and potentially overstepping into domains traditionally navigated by medical science. One area of inquiry in reviews of fatāwa on vaccination is the classification of such actions within Islamic obligations, or whether such actions are even classified at all. Vaccination may be framed by some as a communal obligation (fardh kifayah) when it serves to protect the public, as with diseases that are highly infectious, or an individual obligation (fardh 'ayn) in cases where one's personal health is directly at risk. 

Beyond these obligations, however, there exists the possibility of classifying vaccination as liked or encouraged (mustahab) or disliked or prohibitively disliked or discouraged (makrūh), depending on the level of necessity perceived to fulfill the criteria of Islamic law and the intention with which one is taking it. Differences can be drawn on the nature of reward, or obligation, arising from an act if there is fear of death in not taking a certain medication or vaccination. This perspective raises a critical question: should scholars delve into issuing rulings on preventive health measures like vaccination, considering it a preferable course of action, or merely limit their guidance to its legality (halal or haram)? Some scholars, such as Sheikh Abdul Aziz Ibn Baz, have endorsed vaccinations as forms of defense against potential harm, but this endorsement does not explicitly recommend the act of taking a vaccine as liked or encouraged (mustahab), rather leaving it as an individual choice so long as it aligns with Sharia's principles. Thus, the boundaries between recommendation and obligation remain blurred, leaving Muslims to navigate a space where spiritual guidance intersects with medical advice.

The question remains whether assigning a moral status to preventive health measures like vaccination should fall under Islamic legal purview, especially when such measures rest on scientific, rather than moral, grounds. Vaccination aims to prevent harm, an end goal in alignment with Islam's ethos, but assigning gradations and moral worthiness to preventive measures differs from promoting treatment for existing ailments. This proactive approach, without the certainty of disease presence, diverges from reactive frameworks that many fatwas address. 

Could a fatwa recommending vaccinations implicitly overextend the scope of Islamic law, moving beyond simply adjudicating the vaccine's permissibility? Or could a formal endorsement of preventive measures risk conflating religious authority with medical expertise, thereby influencing Muslims to view vaccination as religiously binding without adequately assessing personal health needs? Such concerns are valid, as there remains an intent for scholars to be grounded in offering moral, not medical, guidance. The question here is one of Islamic jurisprudence potentially extending beyond its customary domain, advising on anticipatory measures whose outcomes are not assured. Health professionals may be better equipped to handle the question of efficacy and necessity, though some Islamic scholars, as well as Muslim physicians, have attempted to assign a status to the act. For example, the Fatwa Committee of Perlis in Malaysia in 2016 declared that it is obligatory (wajib) for parents to vaccinate their children against certain infectious diseases, such as influenza and measles, to name a few. A statement by the International Islamic Fiqh Academy declared that it was a duty upon parents to allow the polio vaccine to be administered to their children, and that not doing so would indicate neglect and potentially a sin due to enabling a causal agent for harm.

There is a potential risk of overturning a religious framework to meet a public health end-goal. When public health institutions reconfigure theological norms into mere instruments of policy, they risk reducing a living moral tradition to a utilitarian tool, thereby eroding both doctrinal integrity and the community trust on which compliance ultimately depends. The fierce resistance that followed Britain's nineteenth-century smallpox vaccination laws, which forced Parliament to insert a conscience clause for objecting parents, is a case showing how compulsory measures can unravel once believers perceive that sacred autonomy has been compromised. Durable public-health gains emerge not from subordinating religion to science, but from negotiating their boundaries in genuine partnership.

It is important to approach public health messaging with sensitivity, especially when addressing religious communities. Suggesting that Muslims who choose not to follow certain public health measures are acting against their faith risks oversimplifying and misrepresenting religious teachings. Additionally, attempts by public health authorities to reshape religious principles to align more closely with their goals raise serious ethical concerns in their own right. Such efforts not only undermine the autonomy of faith groups to regulate their religious beliefs and practices but may also serve to overstep values such as informed decision-making, respect for individual and communal autonomy, and the acceptance of diverse worldviews. Faith traditions may not prioritize a certain conceptualization of health as the ultimate value or end-goal, and co-opting religious narratives to serve health agendas disregards this diversity of perspective. The diversity of thought that exists within Islamic scholarship on the issue, and distinguishing that from the opinions of Muslim physicians, public health experts, or laypersons with respect to this domain, is imperative to constructing guidance in these situations.

Ultimately, preventive health represents a unique intersection where religious and medical ethics converge yet diverge in function. While fatwas on the legality of vaccine components provide clear direction, the prescriptive role of scholars in preventive health remains an evolving field, one that calls for nuanced deliberation. Addressing this evolving role requires balancing respect for religious authority with the recognition of scientific boundaries, ensuring that the preventive health advice issued by religious or public health authorities recognizes the distinction of the domains they are operating within. A plausible path forward is a co-governance model that seats qualified jurists, practicing clinicians, and public health scientists on standing advisory councils charged with issuing joint opinions on emerging interventions. Councils would deliberate with transparent minutes, require dual evidentiary thresholds, establish clinical efficacy and jurisprudential consistency with Islamic jurisprudence, and mandate periodic review when either the scientific data or the socio-religious context shifts. Liaison officers drawn from medical associations would provide scholars with up-to-date pharmacological dossiers, while seminar series in major teaching hospitals would familiarize clinicians with the logic of Islamic legal reasoning, cultivating reciprocal literacy that prevents disciplinary encroachment. By institutionalizing mutual consultation rather than episodic outreach, such a framework preserves doctrinal integrity, enhances public trust, and ensures that preventive guidance remains both theologically sound and scientifically robust.
